# Antimicrobial Peptides-Loaded Hydroxyapatite Microsphere With Different Hierarchical Structures for Enhanced Drug Loading, Sustained Release and Antibacterial Activity

**DOI:** 10.3389/fchem.2021.747665

**Published:** 2021-10-14

**Authors:** Dandan Hong, Jingjing Wu, Xuemin Xiao, Xueyang Li, Dong Xu, Chang Du

**Affiliations:** ^1^ Department of Biomedical Engineering, School of Materials Science and Engineering, South China University of Technology, Guangzhou, China; ^2^ National Engineering Research Center for Tissue Restoration and Reconstruction, South China University of Technology, Guangzhou, China; ^3^ Key Laboratory of Biomedical Materials and Engineering of the Ministry of Education, and Innovation Center for Tissue Restoration and Reconstruction, South China University of Technology, Guangzhou, China; ^4^ Department of Colorectal Surgery, Sixth Affiliated Hospital, Sun Yat-sen University, Guangzhou, China; ^5^ Guangzhou Regenerative Medicine and Health Guangdong Laboratory, Guangzhou, China

**Keywords:** antimicrobial peptides, hierarchical structure, mesoporous carrier, controlled release, hydroxyapatite microspheres

## Abstract

Antimicrobial peptides (AMPs) have great potential for clinical treatment of bacterial infection due to the broad-spectrum and highly effective antibacterial activity. However, the easy degradation and inactivation *in vivo* has been a major obstacle for their application and an effective delivery system is demanding. The surface physicochemical properties of the carrier, including surface potential, surface polarity, pore structure and morphology, have exerted great effects on the adsorption and release behavior of AMPs. This study investigated the influence of micro/nano carriers with different hierarchical structures on the loading, release and biological behavior of AMPs. Three types of AMPs-loaded hydroxyapatite microspheres (HA/AMPs MSs) with different hierarchical structures (needle-like, rod-like, and flake-like) were developed, which was investigated by the surface morphology, chemical composition and surface potential in detail. The different hierarchical structures of hydroxyapatite microspheres (HA MSs) had noticeable impact on the loading and release behavior of AMPs, and the flake-like HA MSs with hierarchical structure showed the highest loading efficiency and long-lasting release over 9 days. Meanwhile, the stability of AMPs released from HA MSs was effectively maintained. Moreover, the antibacterial test indicated that the flake-like HA/AMPs MSs showed more sustained antibacterial properties among three composites. In view of the excellent biocompatibility and osteogenic property, high loading efficiency and the long-term release properties of HA MSs with hierarchical structure, the HA/AMPs MSs have a great potential in bone tissue engineering.

## Introduction

Biomaterial associated infection (BAI) remains an admittedly global problem, especially in orthopaedic surgery (Wang et al., 2020). This infection may usually occur in the initial stage after surgery. It has severely affected the optimal function of dental repairs, bone cements and medical device (Jiao et al., 2017). With the rise of bacterial adaptability, the effectiveness of traditional antibiotics is increasingly threatened (Salas-Ambrosio et al., 2021). One preferred solution for infection prevention is in virtue of the non-traditional antibacterial agents, such as cationic antimicrobial peptides (AMPs). AMPs widely exist in living organisms and they are the important part of immune system. Moreover, AMPs have low toxicity and immunogenicity, little probability of developing resistance and rapid bactericidal activity against a wide range of bacteria (Andersson et al., 2016). However, AMPs is easily degraded and inactivated in human body fluids due to the proteolysis of enzymes or high ionic strength, which lead to its limited application *in vivo* (Mourtada et al., 2019). For orthopedic surgery, the local delivery of AMPs is an ideal solution for the peri-implant infection treatment with reference to a higher antimicrobial efficiency, a lower probability for bacterial resistance, and a better control of antimicrobial distribution to avoid systemic toxicity (Nordstrom and Malmsten, 2017). An important challenge in this strategy was to develop a desired AMPs delivery system that could be efficiently integrated with drugs through physical or chemical means to ensure the bioactivity and achieve controlled release of cargoes (Borro and Malmsten, 2019; Yang et al., 2019; Song et al., 2021).

The covalent modification of AMPs on substrate materials is a common strategy for local delivery (Boden et al., 2018; Chouirfa et al., 2019; Acosta et al., 2020). This method requires relatively complicated processing steps, which may bring about changes in the primary and/or secondary structure of AMPs (Chen et al., 2021). Moreover, the covalently immobilized AMPs have poor stability to enzymes and low activity against the inaccessible bacteria in surrounding tissues (Aveyard et al., 2017; De Zoysa and Sarojini, 2017). On the contrary, the local delivery of AMPs *via* physical effect with the substrate has the features of a simple process, no structural design, better protection of the AMPs from degradation peptides, adjustable release kinetics and easy access to bacteria around the tissues (Kazemzadeh-Narbat et al., 2013; Yu et al., 2020). Studies have shown that the surface potential, specific surface area, pore structure and surface polarity of substrate had noticeable impact on the adsorption and release behavior of cargoes (Braun et al., 2016; Ye et al., 2020; Zou et al., 2021). For instance, the surface polarity of self-assembled monolayers on substrates has recently been shown to be the dominant factor in mediating the interactions of substrate with GL13K supramolecular amphiphiles (Ye et al., 2020). Moreover, the surface morphology of substrate could regulate the osteogenic differentiation of stem cells, which has also exhibited remarkable influence on the adsorption and release behavior of drug molecule (Huang et al., 2018; Jiang et al., 2018). For instance, the particles with hierarchical or hollow structure could significantly increase drug loading compared with solid HA particles (Li J. et al., 2016; Deloney et al., 2020). Li et al. has shown that the hybrid hierarchical CaCO_3_/rGO microspheres presented better doxorubicin loading capacity and sustained release property of the drugs compared with that of CaCO_3_/rGO cube bulks and CaCO_3_/rGO solid spheres (Li J. et al., 2016). Nevertheless, the effects of different hierarchical structures of carriers on the adsorption and release of AMPs have been rarely reported so far.

Hydroxyapatite has shown great promise as drug delivery systems for bone-related diseases in view of its good biocompatibility, low immunogenicity, pH-dependent degradation and excellent osteogenic activity (Li D. et al., 2016; Jin et al., 2019; Li et al., 2020). By means of chemical deposition, emulsion, template or hydrothermal methods, hydroxyapatite with a controllable morphology, pore structure and high specific surface area can be obtained. In our previous work, we synthesized a series of biomimetic hydroxyapatite micro/nano particles with tailorable hierarchical structures through a hydrothermal process and suggested that the flake-like hierarchical structure of HA MSs could more significantly promote osteogenic differentiation of stem cells (Xu et al., 2020). Herein, HHC36 (KRWWKWWRR), one of the most potent broad-spectrum AMPs (Kazemzadeh-Narbat et al., 2013), was selected to evaluate the loading and release behavior of different HA MSs with hierarchical structure and the long-term antibacterial properties of HA/AMPs MSs. The physicochemical features of HA/AMPs MSs were investigated in detail to confirm the efficient integration of AMPs on different HA MSs. The circular dichroism and mass spectrometry were utilized to reveal the stability of the AMPs released from HA MSs. Moreover, the adsorption and release behavior of AMPs on different HA MSs with hierarchical structure, and the long-term antibacterial activity of HA/AMPs MSs were further studied.

## Materials and Methods

### Materials

Antimicrobial peptide HHC36 (KRWWKWWRR) was obtained from Qiangyao Biotechnology Co., Ltd. (purity: 99%, Shanghai, China). (NH_4_)_2_HPO_4_ and Ca(NO_3_)_2_·4H_2_O were purchased from Guangzhou Chemical Reagent Factory (China). DMEM medium, Fetal Bovine Serum (FBS) were purchased from Gibco BRL (Gaithersburg, MD, United States). *Escherichia coli* (*E. coli*, ATCC 8739) and *Staphylococcus aureus* (*S. aureus*, ATCC 6538) were provided by Guangdong Culture Collection Center (China). Nutrient broth and LB agar were purchased from Huankai Microbial Sci. and Tech. Co., Ltd. (Guangdong, China).

### Preparation of HA MSs With Different Hierarchical Structures

The different HA MSs were prepared by a hydrothermal process according to our previous work (Xu et al., 2020). The aqueous solution of (NH_4_)_2_HPO_4_ (2 mM) was prepared and the pH was adjusted to 6.0. Ca (NO_3_)_2_·4H_2_O (3.3 mM) was added to the solution, and the pH was further adjusted to 5.0. Then sodium citrate (0.5 mmol) was added to the above solution under vigorously stirring. And the prepared solution was transferred to a Teflon-lined autoclave to react for 3 h at 180°C. Then the obtained precipitates were centrifuged and freeze-dried, and the sample was named A1 microspheres (A1 MSs). After increasing the concentration of the mixed solution to 3 times, the obtained sample by the same method was named A2 microspheres (A2 MSs). Similarly, when increasing the concentration to 12 times, sample A3 microspheres (A3 MSs) was obtained.

### The Adsorption Behavior of AMPs Onto the HA MSs

The HA MSs at 1 mg/ml were immersed in 1 mM of AMPs solutions, and then incubated on a shaker at 37°C for 2 h. Then the samples were centrifuged and freeze-dried. The obtained samples were named A1/AMPs MSs, A2/AMPs MSs and A3/AMPs MSs, respectively. The amount of peptides in supernatant were analyzed by Thermo Scientific Microplate Reader (Varioskan Flash3001, United States) with a standard curve of AMPs (λ_ex_ = 280 nm). The AMPs loading capacity of HA MSs was evaluated by drug loading rate (DLR) as the following equations.

### Surface Morphology of HA MSs and HA/AMPs MSs

The surface morphology of the HA MSs and HA/AMPs MSs was characterized by a field-emission scanning electron microscope (FE-SEM, Nova Nano SEM 430). The samples were sputter-coated with platinum for 60 s and observed at an accelerating voltage of 5 kV.

### The Physicochemical Properties of HA/AMPs MSs

The distribution of AMPs on HA MSs was visualized by a Laser Scanning Confocal Microscope (LSCM, Leica TCS SP8). The AMPs modified with FITC molecules (FITC-AMPs) were laden on the representative HA MSs. The chemical bond structures of sample were analyzed by Fourier transform infrared spectroscopy (FTIR, Bruker Vector 33 FTIR spectrometer) on KBr pellets in the range of 4,000–400 cm^−1^. The zeta potential of sample was measured by the Zetasizer Nano ZS (Malvern Instruments, United Kingdom). The sample solution with good dispersibility was obtained by ultrasound for 5 min. The chemical composition of sample was detected by X-ray photoelectron spectroscopy (XPS, Thermo Fisher Scientific, ESCALAB Xi+) using a monochromatic Al Kα radiation with a power of 150 W and a 650 µm beam spot.

### Drug Release of AMPs From the HA MSs

The release behavior of AMPs from different HA MSs was investigated in detail. 1 mg samples were immersed in 1 ml PBS at 37°C, then 0.25 ml release medium was withdrawn at different time point and replaced with 0.25 ml fresh PBS. The collected medium was detected for AMPs contents, and each test was performed in triplicate.

### The Stability Assessment of Released AMPs

The structural stability of released AMPs was assessed by the secondary structure and molecular weight. The secondary structure of AMPs released from HA MSs at 1, 4 and 7 days was studied by circular dichroism (CD, Chirascan Spectrometer, CS30320, United Kingdom). The spectra were recorded in the range of 190–260 nm with a step size of 1.0 nm and a bandwidth of 1.0 nm. The molecular weight of released AMPs was tested by a liquid chromatography - mass spectrometry (LC-MS, Waters ZQ 2000) in electrospray mode.

### The Biocompatibility of HA/AMPs MSs

The biocompatibility of different HA/AMPs MSs was evaluated by MTT assay. The mBMSCs were cultured with DMEM containing 10% FBS in an incubator at 37°C under a 5% CO_2_ atmosphere. Cells were seeded into a 96-well plate at a density of 3,000 per well. After cell adherence for 24 h, the culture medium was updated with the extracts of HA/AMPs MSs every 2 days. The control group was cultured with DMEM containing 10% FBS at the same conditions. After culture for 24 and 72 h respectively, the mBMSCs viability was tested by MTT assay kit.

### Antibacterial Properties of HA/AMPs MSs

*E. coli* and *S. aureus* were chosen to evaluate the antibacterial properties of HA/AMPs MSs with different hierarchical structure. The samples was mixed with bacterial solution at a final concentration of 10^5^ CFU/ml. The mixed solution was incubated in a shaker at 37°C for 2 h. Then the above solution was diluted with PBS for the assessment of the bacterial viability on agar plates. Moreover, the morphology of bacteria treated with samples for 2 h was observed by SEM. The bacteria solution was fixed with paraformaldehyde and then dehydrated with ethanol solution. 50 µL of the dehydrated bacterial sample was dropped on the silicon wafer, air-dried naturally and sputter-coated with platinum for 60 s. Furthermore, the extracts of different composite microspheres were used to evaluate their continuous antibacterial activity. At each time point, half of the sample extract was taken for antibacterial activity detection and supplemented with the equal PBS.

### Statistical Analysis

The data were presented as mean ± standard deviation. Statistical analysis was performed using the one-way Analysis of Variance (ANOVA).

## Results and Discussion

### Morphology and Structure of HA MSs and HA/AMPs MSs

**FIGURE 1:**
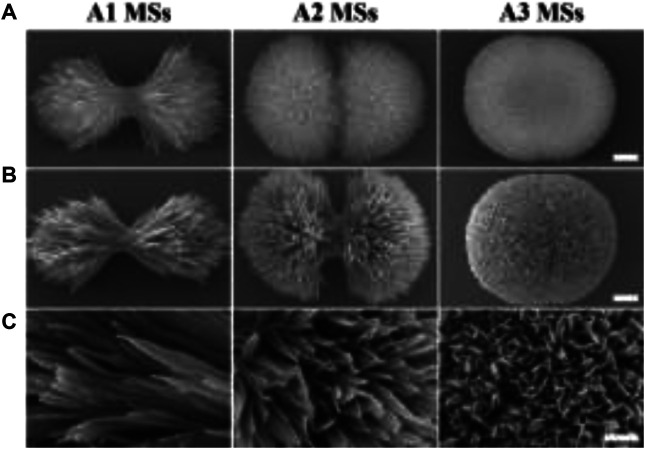
The morphology of HA MSs and HA/AMPs MSs. **(A)** Morphology of diverse HA MSs, bar = 0.5 µm. **(B)** Morphology of different HA/AMPs MSs, bar = 0.5 µm. **(C)** Magnified images of the surface topography of HA/AMPs MSs, bar = 0.2 µm.

The success of bone defect repair depends not only on the effective osseointegration of substitute with bone tissue, but also on the presence of a sterile environment around the substitute (Karadjian et al., 2019; Jacobs et al., 2020). Many studies have shown that the micro/nano morphology of substrate could prominently promote cell adhesion, cell proliferation, immune response and osteogenic differentiation (Jiang et al., 2018; Zhao et al., 2018). Particularly, the micro/nano structure and high specific surface of micro/nano materials seasonably contribute to the integration of antibacterial agent for preventing bacterial infections during orthopedic surgery. However, the absorption and release behavior of AMPs on the different micro/nano hierarchical structures of substitute was still undiscovered. To study the influence of HA microspheres with different hierarchical structures on the adsorption and release behavior of AMPs, three types of HA MSs (needle-like, rod-like, and flake-like) were prepared by a hydrothermal process mediated by citrate. When the concentration of reaction system increased from 3 times to 12 times, the morphology of HA particles developed from microflowers to microspheres (Figure 1A). The different particles presented hierarchical micro/nano-scale surface textures evolving from a needle-like to a rod-like, until a flake-like. Apparently, when AMPs was absorbed onto the particles, the morphology of the different HA MSs had no distinct change (Figure 1B). It was confirmed from a more microscopic structure in Figure 1C.

### The Physicochemical Properties of HA MSs and HA/AMPs MSs

**FIGURE 2:**
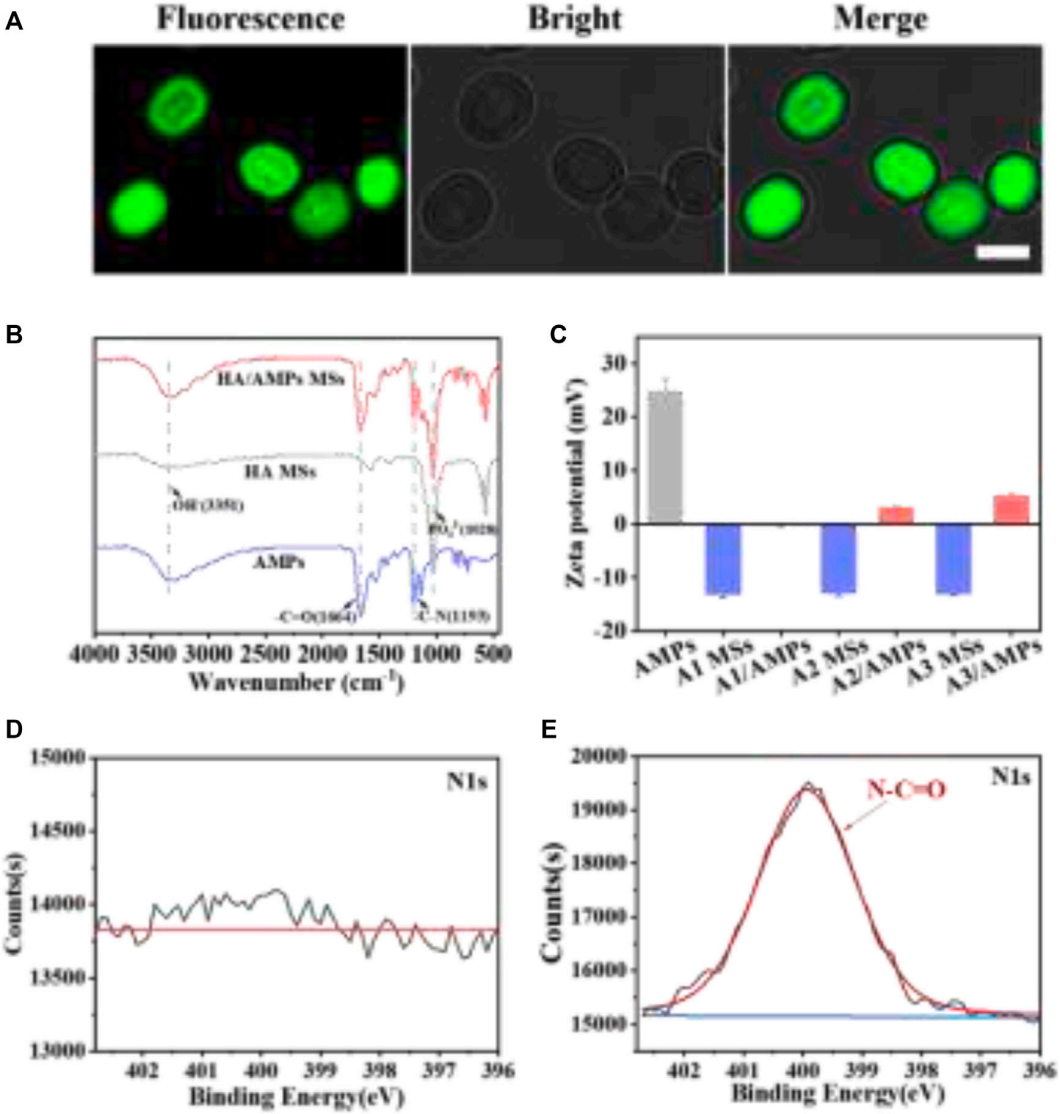
The physicochemical properties of composite microspheres. **(A)** Visualized distribution of FITC-AMPs on representative HA MSs by LSCM, bar = 2 µm. **(B)** FTIR spectra of representative HA MSs and HA/AMPs MSs. **(C)** Analysis of zeta potential. **(D,E)** N1s spectra of HA MSs and HA/AMPs MSs by XPS.

In order to affirm that AMPs were loaded on HA MSs, the visual distribution of FITC-AMPs on the microspheres was observed by LSCM (Figure 2A). The HA MSs presented positive fluorescence emission, which more intuitively illustrated the successful encapsulation of AMPs on the HA MSs. And the other two HA/AMPs MSs also presented obvious fluorescence phenomenon. The characteristic spectral bands of AMPs around 1,664 cm^−1^ and 1,193 cm^−1^ in the FTIR pattern were assigned to vibration of -C=O and -C-N (Figure 2B). The characteristic spectral band around 3,351 cm^−1^ was ascribed to the vibration of OH^**−**^, and 1,028 cm^−1^ was described to PO_4_^3-^. These characteristic spectral bands also occurred in HA/AMPs MSs, which indicated the effective integration of HA MSs and AMPs.

**FIGURE 3:**
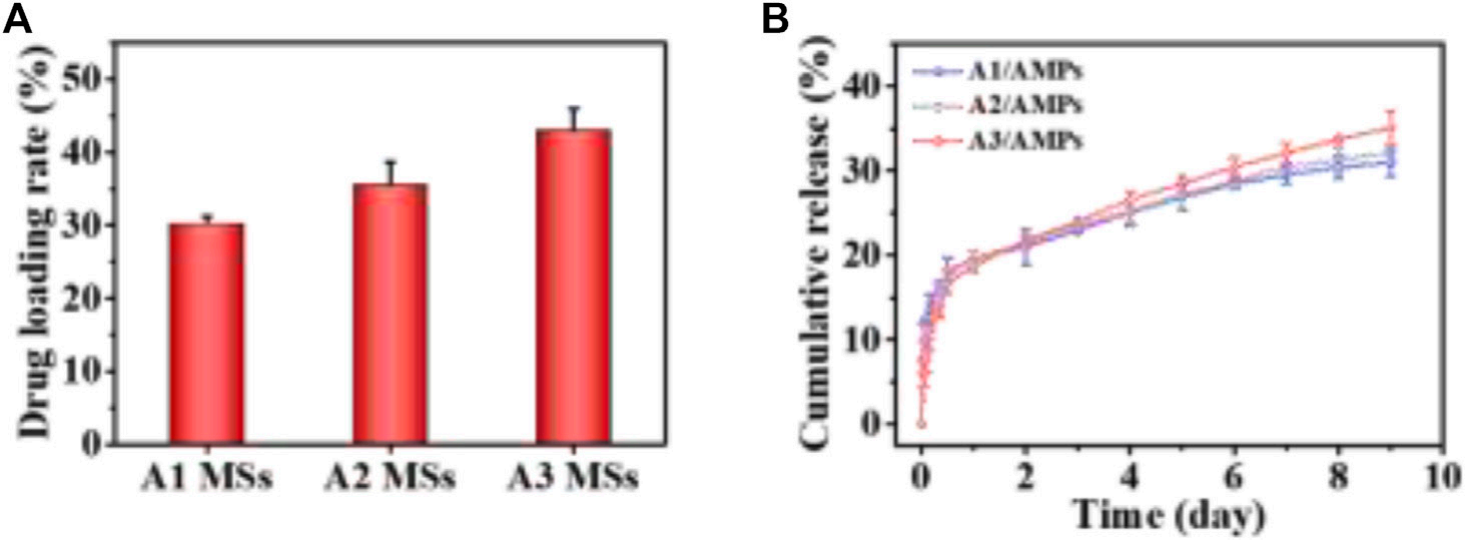
The loading and release property of AMPs from HA MSs. **(A)** Drug loading property of different HA MSs for AMPs. **(B)** release profile of AMPs from different HA MSs.

Moreover, the surface potential of different samples was evaluated (Figure 3C). The zeta potential of three different HA MSs was similar and that of AMPs was 24.8 ± 2.29 mV. While the potential of different composite microspheres were -0.21 ± 0.09, 2.96 ± 0.15, and 5.22 ± 0.19 mV respectively, which could be attribute to high loading capacity of AMPs on different HA MSs. The chemical composition of HA/AMPs MSs was further investigated in detail (Figures 2D,E). The N-C=O peaks of HA/AMPs MSs at 400 eV appeared and became the major component of the high-resolution N1s spectra (Figure 2E). While no evident N1s peak occurred in HA MSs. These results further confirmed the combination of HA MSs with AMPs.

### AMPs Loading Capacity and Release Kinetics

The diverse crystallite texture and mesoporosity of HA MSs could dramatically influence the specific surface area and then their drug loading capacity. HHC36 was selected as the model molecule for the AMPs loading study due to its broad-spectrum and highly effective antimicrobial activity (Kazemzadeh-Narbat et al., 2013; Andersson et al., 2016). The drug loading rate of A3 MSs for AMPs was 42.93 ± 3.11%, which was significantly higher than that of other two microspheres (Figure 3A). The increased trend in the AMPs loading capacity of three different HA MPs with hierarchical structure was consistent with that of their specific surface area (Xu et al., 2020). It suggested that the increased specific surface area of HA MSs had significant effect on the loading capacity of AMPs. These analyses indicated that the electrostatic binding facilitated the adsorption of cationic antimicrobial peptide, which associated with the unique hierarchical structure of micro/nano flake-like HA MSs remarkably promoted the adsorption of AMPs.

Moreover, the releasing of AMPs from different HA MSs with hierarchical structure was investigated in PBS at 37°C, as presented in Figure 3B. There was an initial burst release for three diverse HA MSs in the first 12 h, which might be ascribed to the loose binding of the about 17.45% AMPs molecules to HA MSs. More than 80% of the loaded AMPs could be tightly bound to the surface and hollow structure of HA MPs through the plentiful mesoporous structure and strong electrostatic interactions. The sudden release of AMPs from A1 MSs was most obvious, which might be attributed to the larger mesoporous in A1 MSs (Xu et al., 2020). It was worth noting that the cumulative release amount of AMPs from A2 MSs was more than that from A1 MSs at 24 h. However, the cumulative amount of released AMPs from A3 MSs had exceeded that from A1 MSs and A2 MSs around 48 h. And a highly sustained release was observed up to 9 days. At this stage, the release profile of AMPs from the HA MPs could be construed as a zero-order release kinetics, which was regarded as the vital merit of delivery systems. The therapeutic molecules could provide a relatively constant dose for several weeks, what was quite essential during the regeneration of bone tissue disease (El-Fiqi et al., 2015).

### The Stability Assessment of the Released AMPs

**FIGURE 4:**
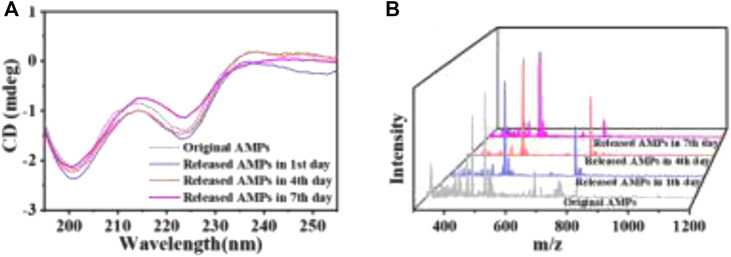
The stability assessment of AMPs released from the representative HA MSs. **(A)** Far-UV CD spectra. **(B)** Mass spectrometry spectra.

To attest the structural stability of the AMPs released from the HA MSs, the secondary structure and molecular weight of the released AMPs were detected by circular dichroism and mass spectrometry. The Far-UV CD spectra of AMPs released from the representative microspheres (A3 MSs) was recorded (Figure 4A). The CD spectrums of released AMPs at different time point were similar with that of original AMPs. Particularly, a strong negative band at 201 nm and a relatively weak negative band at 223 nm occurred in these CD spectrums. These results revealed a relatively stable secondary structure of the released AMPs, which indicated that the mesoporous structure could protect AMPs from chemical and enzymatic degradation and prevent conformational changes and/or peptide aggregation (Nordstrom and Malmsten, 2017). Moreover, the mass spectrometry showed that the molecular weight of released AMPs at 1, 4 and 7 days was 1,488 (Figure 4B), which kept in consistence with that of the original AMPs molecule. The consistency between the secondary structure and molecular weights indicated that the released AMPs maintained a high stability.

### The Biocompatibility of HA/AMPs MSs

**FIGURE 5:**
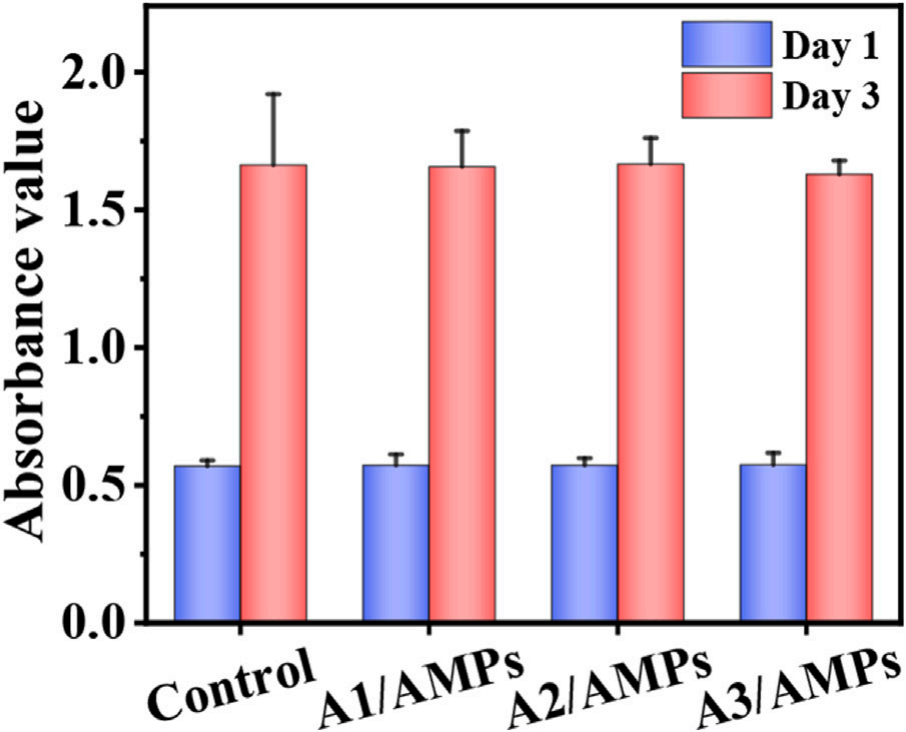
The biocompatibility of the extracts from different HA/AMPs MSs when cultured with mBMSCs, n = 5.

The good biocompatibility is one of the indispensable properties for a drug carrier. It has been shown that three different HA MSs with hierarchical structure had a good biocompatibility within a certain concentration range in our previous work (Xu et al., 2020). In order to evaluate the potential cytotoxicity of HHC36 released from the HA MSs to host tissue cells, the viability of mBMSCs after culture with the extracts of three HA/AMPs MSs at different times was detected by the MTT assay (Figure 5). There was no significant difference (*p* > 0.05) in cell proliferation activity between the composite group and the control group in different time points. However, many studies have shown the potential cytotoxicity of the HHC36 at high concentration on stem cells (Chen et al., 2020; Chen et al., 2021). It indicated that the sustained release effect of HA MSs with hierarchical structure on HHC36 effectively decreased the cytotoxicity to stem cells, which further illustrated the potential applications of composite microspheres in bone tissue engineering.

### Antibacterial Assay

**FIGURE 6:**
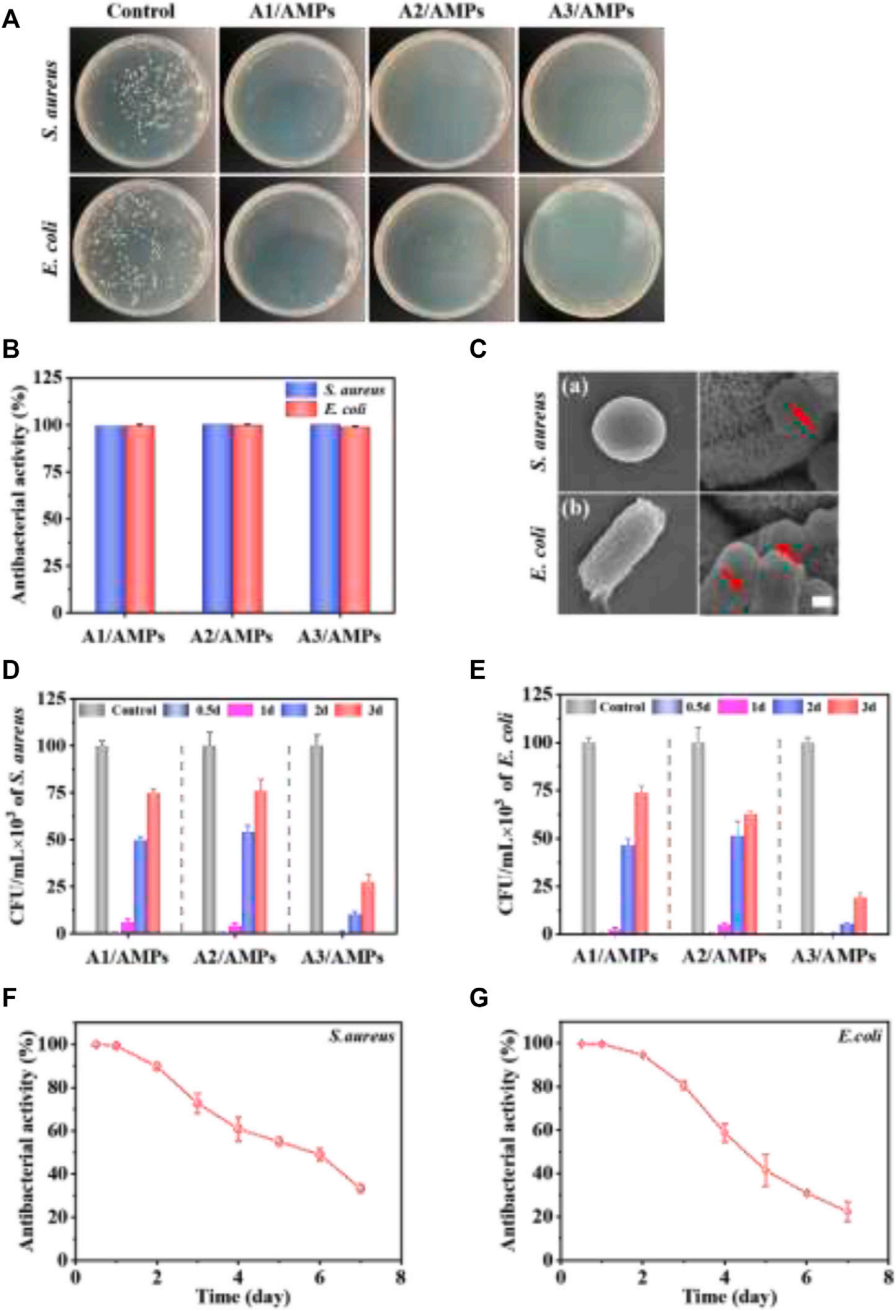
The evaluation of the continuous antibacterial property of different HA/AMPs MSs. **(A)** Bacterial colonies formation after culture with HA/AMPs MSs for 2 h. **(B)** Antibacterial activity of the HA/AMPs MSs at 2 h. **(C)** SEM images showed the changes of bacterial membrane after culture with A3/AMPs MSs for 2h, bar = 0.5 µm. **(D,E)** Antibacterial activity of three HA/AMPs MSs at different time points of 0.5, 1, 2, and 3 days. The control group was cultured with HA MSs extract. **(F,G)** The long-term antibacterial performance of A3/AMPs MSs from 0.5 to 7 days.

The antibacterial property of the HA/AMPs MSs was evaluated by the plate-counting method. Almost no bacteria were observed on the plates in the three HA/AMPs MSs groups after co-culture with *S. aureus* and *E. coli* for 2 h (Figure 6A). The quantitative analysis also confirmed that three HA/AMPs MSs showed nearly 100% bactericidal activity against both negative and positive bacteria within 2 h (Figure 6B). Moreover, the pore formation and membrane rupture were observed on the surface of both bacteria (Figure 6C). The electrostatic interaction of AMPs with the bacterial membrane and the further insertion of its hydrophobic components into the bacterial membrane was generally considered to generate the pore formation and bacterial death (Lee et al., 2016; Li et al., 2021).

To investigate the sustained antibacterial activity of different HA/AMPs MSs mediated by hierarchical structure, *S. aureus* and *E. coli* were co-cultured with their extracts at different time points (Figures 6D,E). The extracts of different composites exhibited about 100% antibacterial activity within 12 h. Furthermore, the antibacterial activity of A3/AMPs MSs extract was 89.76 and 94.69% for *S. aureus* and *E. coli* respectively on the second day instead of the significantly weakened one of the extracts from A1/AMPs MSs and A2/AMPs MSs. However, the extracts from A3/AMPs MSs on the third day still had an obvious inhibitory activity on both bacteria. Although the release behavior of AMPs from A3 MSs showed slight difference with that from A1 MSs and A2 MSs, the drug loading rate of A3 MSs was significantly higher than that of A1 MSs and A2 MSs. These resulted in the more obvious and sustained antibacterial effects of A3/AMPs MSs compared with other composites. To further evaluate the long-term antibacterial property of A3/AMPs MSs, the antibacterial activity of its extracts in the first 7 days was evaluated for both bacteria in detail (Figures 6F,G). The antibacterial activity was about 60% for both bacteria on the fourth day, which indicated that A3/AMPs MSs could show an excellent effect in the early stage of implantation. And the antibacterial activity for *S. aureus* and *E. coli* at the concentration of 10^5^ CFU/ml were still 30 and 22% respectively on the seventh day, which implied a continuous antibacterial effect of A3/AMPs MSs. These results revealed that A3/AMPs MSs exhibited high-efficiency and long-term antibacterial effects, indicating a potential applications in the bone tissue engineering.

## Conclusion

The present study has shown that the adsorption and release of AMPs was strongly influenced by the different hierarchical structures, and the flake-like HA MSs with hierarchical structure showed the highest loading efficiency and long-lasting AMPs release among the three HA MSs. The circular dichroism and mass spectrometry revealed that the AMPs released from HA MSs retained a high stability. On account of the slow release of AMPs from the mesoporous HA MSs, the HA/AMPs MSs exhibited good biocompatibility on stem cells. The plate-counting method and SEM indicated that three types of HA/AMPs MSs showed highly effective antibacterial activity in the early stage. The further analysis revealed that the flake-like HA/AMPs MSs showed more sustained antibacterial effects compared with the other composite microspheres against *S. aureus* and *E. coli*. Our research shed light on the high loading efficiency and the long-term release properties of HA MSs with hierarchical structure, and the great potential of HA/AMPs MSs in the complex treatment conditions of bone tissue diseases.

## Data Availability

The original contributions presented in the study are included in the article/supplementary material, further inquiries can be directed to the corresponding authors.
